# A Review of Dietary* Ziziphus jujuba* Fruit (Jujube): Developing Health Food Supplements for Brain Protection

**DOI:** 10.1155/2017/3019568

**Published:** 2017-06-07

**Authors:** Jianping Chen, Xiaoyan Liu, Zhonggui Li, Airong Qi, Ping Yao, Zhongyu Zhou, Tina T. X. Dong, Karl W. K. Tsim

**Affiliations:** ^1^Shenzhen Key Laboratory of Hospital Chinese Medicine Preparation, Shenzhen Traditional Chinese Medicine Hospital, Guangzhou University of Chinese Medicine, Shenzhen 518033, China; ^2^Division of Life Science, Center for Chinese Medicine, The Hong Kong University of Science and Technology, Clear Water Bay, Hong Kong; ^3^Shenzhen Research Institute, The Hong Kong University of Science and Technology, Shenzhen 518057, China

## Abstract

The fruits of* Ziziphus jujuba*, known as jujube or Chinese date, are being consumed all around the world because of their health benefits, as both food and herbal medicine. Traditionally, one of the main functions of jujube, as described in herbal medicine, is to benefit our brain by calming down the mind and improving quality of sleep. Here, the activities of jujubes on nervous system are summarized and discussed. Jujube possesses neuroprotective activities, including protecting neuronal cells against neurotoxin stress, stimulating neuronal differentiation, increasing expression of neurotrophic factors, and promoting memory and learning. Flavonoid, cAMP, and jujuboside could be the potential bioactive ingredients to account for the aforesaid biological activities. These findings imply that jujube is a potential candidate for development of health supplements for prevention and/or treatment of neurological diseases.

## 1. Introduction

**Figure 1:**
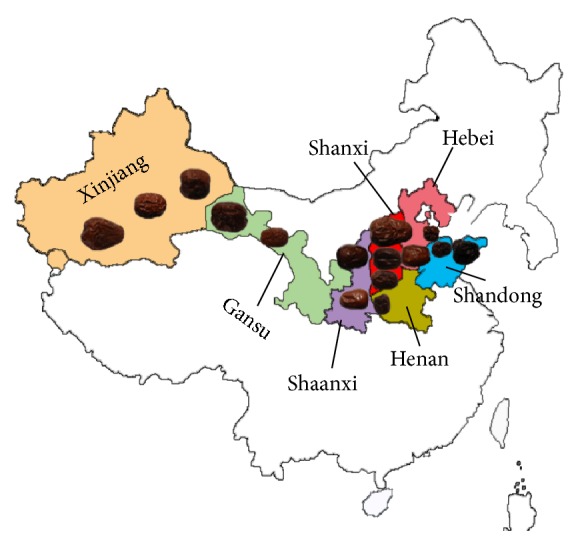
*The cultivation areas and major production areas of jujube in China*. The cultivation areas of jujube in China were highlighted and shown. The insert shows the location of major production areas of China, including Xinjiang, Gansu, Shaanxi, Shanxi, Hebei, Henan, and Shandong provinces. Shaanxi and Shanxi are considered as the original cultivation areas of jujube.

Jujubae Fructus, the fruit of* Ziziphus jujuba* Mill. (Rhamnaceae), also known as jujube, or Chinese date, or red date, has been widely used as food and Chinese herbal medicine for over 3,000 years. Jujube is indigenous to Chinese culture (Figure 1). The description of jujube was first recorded in Classic of Poetry (1046-771 BC). Today, there is a wide distribution of jujube-related products in the world. The consideration of jujube as a vital food and/or medicine has a long history of record in China. In ancient Chinese book on herbal medicine* Huangdi Neijing* (475-221 BC), jujube was described as one of the five most valuable fruits in China. In* Shennong Bencao Jing* (300 BC-200 AD), an earlier book recoding medicinal herbs, jujube was considered as one of the superior herbal medicines that prolonged our life-span by nourishing blood, improving quality of sleep, and regulating digestive system.

Jujube has been consumed for thousands of years, which is still gaining influence on our daily life. Recent phytochemical and pharmacological results have revealed that flavonoid, polysaccharide, and triterpenic acid are the main active ingredients within jujube. Based on the literatures, both flavonoid and polysaccharide could account for antioxidative effect of jujube [1–3]. Moreover, jujube polysaccharides were also proposed to be main active ingredients contributing to its immune-modulating and hematopoietic functions [4, 5]. Triterpenic acids were considered as active ingredients for the effect on anti-inflammatory and anticancer activities [6, 7]. In addition, betulinic acid and jujuboside B could be the active components showing beneficial effects on cardiovascular system [8, 9].

The study on biological activities has supported the health benefits of jujube as both food and medicinal herb. According to Chinese medicinal theory, jujube is considered as a medicinal herb that calms the mind and relieves mental tension. Clinically, jujube is commonly prescribed, either as single herb or in tranquillizing formulae combined with other herbal medicines, for the treatment of insomnia and forgetfulness. Previous reviews have summarized the fruit composition and its health benefits [10, 11]. However, the studies focused on neuroprotective activities are rather limited. Therefore, the neurobeneficial roles of jujube will be introduced in this review. In addition, the potential bioactive compounds of jujube related to brain benefits are further discussed.

## 2. Jujube on Neuron Differentiation

**Figure 2:**
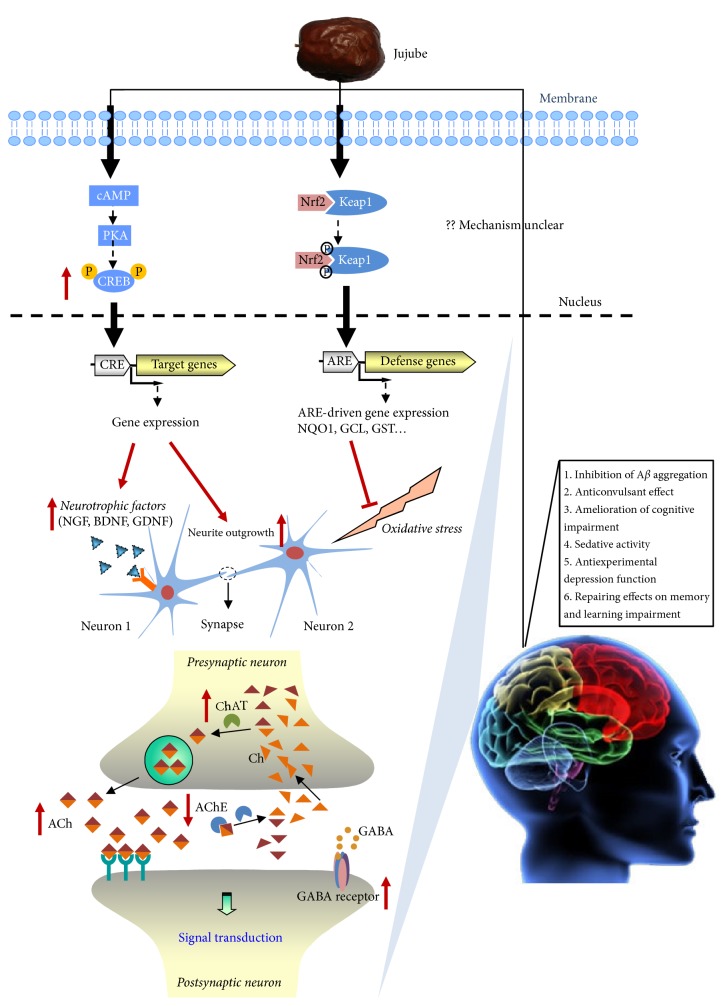
*The neuroprotection effects of jujube*. Jujube induces neurite outgrowth and neurothrophic factor expression via cAMP-dependent PKA signaling. Jujube possessed neuroprotection against oxidative stress via enhancing cellular Nrf2-dependent ARE-driven gene expressions. Jujube improves the choline acetyltransferase (ChAT) activity and shows inhibitory activity against acetylcholinesterase (AChE). Jujube increases the level of acetylcholine (ACh) in the brain. Jujube stimulates the transcriptional expression of GABA receptor subunits in rat hippocampal neurons.

According to historical usage in China, one of the main functions of jujube was considered to benefit our brain by calming down the mind and improving quality of sleep. In modern science, benefiting our brain is usually related to neurobeneficial effects, for example, neuroprotection effect and neurotrophic action. In neurological disorders, for example, neurodegenerative diseases, insomnia, and depression, several common pathological conditions among them are found, that is, neurogenesis impairment, neurotrophic factor deficiency, and oxidative stress. Hence, the traditional function of jujube in benefiting the brain may be closely related to its neurobeneficial effects (Figure 2). Here, the neurobeneficial effects of jujube are summarized (Table 1).

Neurogenesis is a well-orchestrated process consisting of neuronal differentiation, synapse formation, and cell proliferation. Some studies suggested that, in pathological condition of neurodegenerative diseases, various types of neurogenesis could be observed, indicating that neurogenesis might be a compensatory mechanism in neurodegenerative processes [12]. Thus, the promotion of neuronal differentiation could be a means to prevent these diseases. Cultured pheochromocytoma PC12 cells are a pertinent model system for the study of neuronal differentiation [13]. The characters of PC12 cells are very similar to sympathetic neuron system, that is, the characters of neurofilament (NF) expression and neurite outgrowth in responding to challenge of nerve growth factor (NGF). The findings on jujube indicated that application of jujube water extract on cultured PC12 cells for 72 hours could induce cell differentiation: the induced differentiated cell accounted for ~25% of the total cells in cultures. The results also showed that application of jujube water extract in cultured PC12 cells for 72 hours could dose-dependently stimulate the expressions of NF68, NF160, and NF200 [13].

Astrocytes are the most abundant cell in the nervous system. One of its main functions is to synthesize and release neurotrophic factors, that is, NGF, brain-derived neurotrophic factor (BDNF), glial cell line-derived neurotrophic factor (GDNF), neurotrophin 3 (NT3), and NT4/5 [14]. These factors are vital for neuronal survival, growth, and differentiation, and the deficiency of which can cause neurological impairments [15]. Thus, the upregulation of neurotrophic factors can play a positive role in treating neurological diseases. The effect of jujube on neurotrophic factor expression was investigated in cultured astrocytes [16]. The treatment with jujube water extract stimulated the expression of neurotrophic factors in a dose-dependent manner, having the highest induction of ~100% for NGF, 100% for BDNF, 100% for GDNF, and 50% for NT3. For NT4 and NT5, no obvious morphological change was observed in jujube-treated astrocytes.

The signaling of cAMP-PKA-CREB is well known to play a role in neuronal differentiation of PC12 cells [17]. Hence, the involvement of cAMP pathway in jujube-induced neurite outgrowth and neurofilament expression was revealed (Figure 2). The findings showed that jujube-induced neurite outgrowth and neurofilament expressions were attenuated by application of H89 (a cyclic AMP-dependent PKA inhibitor) [13]. CREB, the nuclear transcription factor, has been known to play a role in neuronal differentiation [18]. It was reported that jujube was able to stimulate the phosphorylation of CREB, and its inductive effect could be fully blocked by H89 [13]. Besides, the application of jujube water extract could stimulate the transcriptional activity of CRE. Further studies also showed that pretreatment with H89 significantly blocked jujube-induced neurotrophic factors including NGF, BDNF, and GDNF, indicating the possible involvement of PKA signaling in jujube-induced neurotrophic factor expression [16].

## 3. Neuroprotection against Oxidation Insult

**Figure 3:**
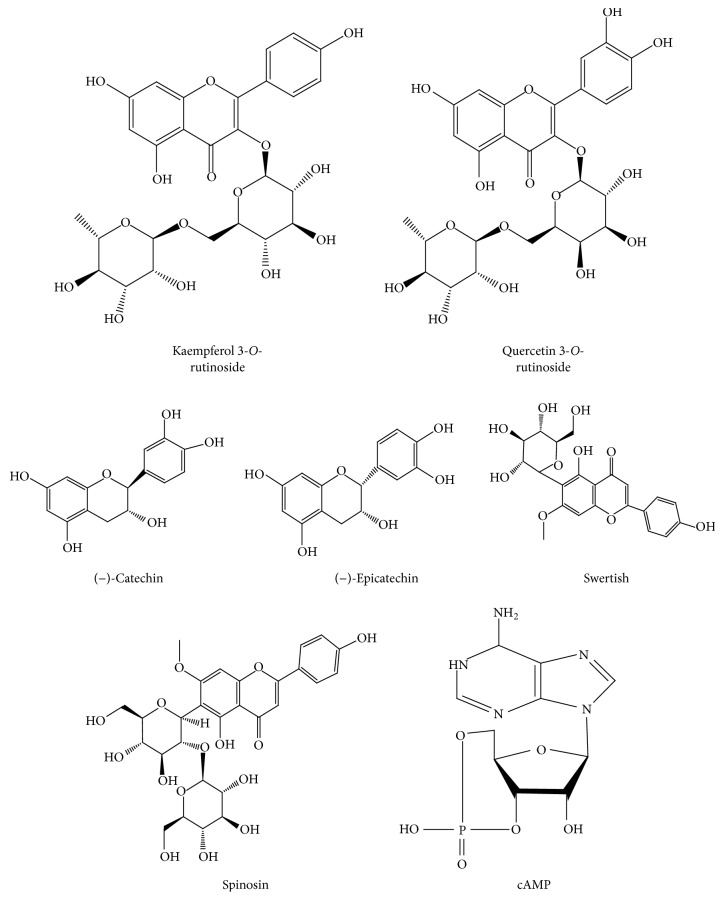
*Structures for chemical compounds in jujube having potential neuroprotection effect*. Seven chemical markers found in jujube including kaempferol 3-*O*-rutinoside, quercetin 3-*O*-rutinoside, (−)-catechin, (−)-epicatechin, swertish, spinosin, and cAMP were reported to possess effect on neuroprotection.

In the process of neurodegeneration, the functions of neuron are markedly decreased. In Parkinson and Alzheimer diseases, oxidative stress, as the main considerable factor, is believed to cause neuronal damage in progress of diseases [29]. In cultured cells, jujube water extract was reported to protect neuronal cells against* tert*-butyl hydroperoxide- (tBHP-) induced oxidative injury. In addition, it was found that jujube water extract could inhibit tBHP-induced ROS (reactive oxygen species) formation in cultured PC12 cells [20]. The Nrf2 (nuclear factor (erythroid-derived 2-) like 2-) dependent ARE (antioxidant response element-) driven genes, including glutamate-cysteine ligase (GCL), glutathione S-transferase (GST), and NAD(P)H quinone oxidoreductase (NQO1), have been demonstrated to play an important role in protecting cells against oxidative stress [30, 31]. Jujube water extracts stimulated the ARE-mediated transcriptional activity, indicating the activation of Nrf2 pathway (Figure 3). Besides, the application of jujube induced the amounts of NQO1, GCLC (catalytic subunit of GCL), GCLM (modifier subunit of GCL), and GST mRNA levels in cultured astrocytes [16]. Jujube was also revealed to protect ischemic damage in gerbil hippocampus via its antioxidant effect, that is, the upregulation of superoxide dismutase (SOD) 1 and reduction of lipid peroxidation [21].

## 4. Jujube on Insomnia, Learning, and Memory

In Chinese herbal medicine, jujube was prepared as a tea that was used against insomnia [32, 33]. Indeed, jujube was found to increase pentobarbital-induced sleep time and to reduce free movement on mice [22]. In support of this, Peng et al. (2000) reported that the seed of* Z. jujuba* prolonged the hexobarbital-induced sleeping time in mice and decreased the locomotor activity in rats. Moreover, jujube was shown to possess anxiolytic effect by increasing the first time entry, as well as the total change and time spent in the white chamber of black and white test [34]. Besides, flavonoids and saponins from seed of* Z. jujuba* showed sedative and hypnotic effects, which caused a significant reduction of walking time and coordinated movement ability of mouse, significantly prolonging its sleeping time [35]. Jujuboside A, one of saponins from seed of* Z. jujuba*, stimulated the expression of GABA receptor subunits in rat hippocampal neurons [36] and ameliorated behavioral disorders of the dementia mouse model induced by A*β*1–42 [37]. Methanolic extract and oleamide from jujube showed activation effect on choline acetyltransferase (ChAT). Meanwhile, oleamide was found to attenuate the scopolamine-induced amnesia in mice, a useful* in vivo* model for Alzheimer disease [26]. Jujube was reported to improve learning and memory in ovariectomized rat model, and the effect of which might be due to an increase of estrogen in the blood, as well as the levels of nitric oxide and acetylcholine in the brain [23]. Moreover, hydroalcoholic extract of jujube was also demonstrated to possess anticonvulsant effect and amelioration of cognitive impairment induced by seizures in rats [24]. The seed extract of* Z. jujuba* showed a promising effect in ameliorating memory in mice with alcohol-induced memory retrieval disorders and might help to improve learning capacity to some extent [38]. In line with the aforementioned reports, jujube was reported to possess repairing effects on memory and learning impairment induced by bilateral electric lesion of the nucleus basalis of Meynert in rats [25]. In addition, jujube extract having 1% of cAMP was found to have antidepression function in animal model [39].

## 5. Two Developmental Stages of Jujube in Neuroprotection

Two types of jujubes are commonly found in China market: fresh immature jujubes were consumed as fruits, and dried mature jujubes were used as Chinese medicinal herb. Chemically, a sharp decrease of flavonoidic compounds was revealed during maturity [20, 40], while the amounts of selected compounds were significantly lower in immature jujubes than that of mature ones, including xanthine, hypoxanthine, adenine, uridine, guanosine, cGMP, and cAMP [20]. Moreover, the metabolite variations between mature and immature jujubes were also studied. Levels of isoleucine, threonine, acetate, creatine, and glucose were lower in immature jujube than those of mature one, while levels of alanine, sucrose, and formate were lower in mature jujube than those of immature one [19]. For biological assessments, the antioxidative functions of jujube extracts were first compared [20]. The antioxidative effects were varied amongst different maturity jujubes. The fresh immature jujube had better effect on antioxidation activity than that of mature one. However, mature jujube showed better effect in inhibition of ROS formation and activation of Nrf2-antioxidant response element signaling [20]. Furthermore, in terms of neuronal differentiation, mature jujube possessed better effect, about 60% higher than that of immature one [19]. These findings suggested the specific usage of different maturity of jujubes.

## 6. Jujube-Containing Herbal Decoctions on Neuroprotection

In Chinese herbal medicine, jujube is commonly included in herbal mixture, called* Fu Fang*, that is having specific combination of different herbs. These specific requirements of herbal preparation have not been changed. Among thousands of different herbal decoctions,* Ganmai Dazao Tang* and* Chaihu Guizhi Tang* are commonly used today for the treatment of depressive disorders [41]: both of them contain jujube as a key herb within the formulation. Taking* Ganmai Dazao Tang* as an example, this prescription having licorice, wheat, and jujube was recorded in a book* Jingui Yaolue* (Jingui Collection of Prescriptions), written by a well-known Chinese medicine scholar Zhang Zhongjing (150 BC to 219 AD) in Han Dynasty of China. This herbal decoction was traditionally prescribed for women suffering from anxiety. Ganmai Dazao Tang was reported to be one of top 10 Chinese herbal formulae prescribed for insomnia in Taiwan [42]. Although the molecular mechanisms for these herbal formulae are unclear, these antidepressive formulae may possess effect on neurotrophic factor expression, as it is well believed that reduction of BDNF expression is observed in depression patients [43, 44]. Indeed, Ganmai Dazao Tang was reported to induce BDNF expression in depressive animal model [45]. Besides, the jujube-containing herbal decoctions, including Guizhi Tang, Neibu Dangguijianzhong Tang, and ZaoTang, induced neuronal differentiation and expressions of antioxidant enzymes in cultured PC12 cells [46], for example, GCLC, GCLM, GST, and NQO1 via the activation of ARE. Together with the result of jujube as single herb alone, these findings could illustrate the functions of jujube within a multiherbal decoction.

## 7. Potential Bioactive Ingredients on Neuroprotection

Phenolic compounds are widely found in plants, which comprise a large group of bioactive ingredients. Flavonoids are commonly found in fruits, and some of which have been reported to possess neuroprotective effect (Figure 3) [47, 48]. Among different types of flavonoids in jujube, Zhu et al. (2007) reported that kaempferol 3-*O*-rutinoside possessed neuroprotective activity against oxidation insult and prevention of A*β* aggregation [47]. Quercetin 3-*O*-rutinoside was shown to have anti-A*β* aggregation effect. Both (−)-catechin and (−)-epicatechin showed inhibition of A*β* aggregation and A*β*-induced toxicity [47]. In addition, kaempferol 3-*O*-rutinoside showed protective effect on permanent focal cerebral ischemia and on neuronal cultures [49], as well as in reducing memory dysfunction, energy metabolism failure, and oxidative stress in multi-infarct dementia model rats [50]. Spinosin possessed potentiating effect on pentobarbital-induced sleep, and the effect of which was related to the postsynaptic 5-HT receptor [51, 52]. Indeed, spinosin and swertish, isolated from jujube seeds, were reported to possess significant sedative activity [53]. Yet, the flavonoids within jujube, including (−)-catechin, (−)-epicatechin, kaempferol 3-O-rutinoside, and quercetin 3-O-rutinoside, were tested for their neurotrophic action, yet no significant effect was found among them [48].

Cyclic nucleotides and their derivatives participate in the regulation and modulation of lots of physiological processes in body. These chemicals exhibit multiple bioactivities, that is, neuroprotective effects [54, 55]. Among them, cAMP was reported to be a relative large amount in jujube, and interestingly its content was much higher than other horticultural fruits (Figure 3) [56–58]. The amount of cAMP, at approximately the amount containing within 2 mg/mL of jujube water extract, showed the activity on neuronal differentiation in cultured PC12 cells. This firmly indicated that jujube cAMP exerted a role in neuronal differentiation, as reported for jujube extract [13]. Besides, a report showed that jujube was able to increase the cAMP content in plasma and hippocampus of animal model [27], and the authors hypothesized that the exogenous cAMP in jujube water extract might induce the aforesaid results. In parallel, cAMP is well believed to be involved in formation of depression. Indeed, cAMP isolated from jujube was found to possess antimelancholic property in animal model of depression [39]. In addition, Jujubosides, found in Semen Ziziphi Spinosae and jujube [59], were reported to possess hypnotic effects [35, 60]. To support this, jujuboside A could adjust GABA receptor subunit mRNAs expression in hippocampal neurons [61]. Oleamide from jujube reversed the scopolamine-induced memory and/or cognitive impairment in mice model [26]. In contrast, a polypeptide snakin-Z, isolated from jujube, possessed cholinesterase inhibitory effect [28].

## 8. Development and Perspective

Having over 3,000 years of history, jujube is still a popular fruit in our daily life for its health benefits [62]. Based on the aforementioned cellular and animal findings, jujube has a great potential in developing further as food and medicinal supplements for brain health. Jujube water extract is the most common usage form. It can be prepared into decoction and drunk daily, or it can be combined with other foods for the preparation of a delicious soup. Clinical studies showed that no adverse and drug interactions have been reported for the consumption of jujube [63–66]. In addition, the recent studies also revealed that jujube water extract contained higher amount of ingredients as compared to that of ethanol extract [20]. Hence, jujube water extract could be a good choice as an alternative to be prescribed for neurological disorder. Moreover, the quality of herbal medicine, as jujube here, could be varied from each other due to numerous factors, for example, harvest season, geographic region, and postharvest treatment. Thus, it is essential to establish different parameters in receiving chemical standardized jujube water extract. In order to chemically standardize jujube water extract in terms of its HPLC profile and chemical contents, the HPLC fingerprint and quantification methods were employed to reveal its HPLC profile and quantify the main ingredients [20]. The standardization parameters can be employed to ensure the consistent quality of jujube water extract and to further make sure of its repeatability of experimental results.

The active ingredients of jujube include nucleotide and flavonoid, which may show benefit effects in brain functions. In consideration of the benefit of cAMP in jujube, numerous studies are targeting on the processes in extracting and separating cAMP from jujube [39, 67, 68]. Hence, the enrichment of jujube nucleotide may be developed into a food or medicinal product for the treatment of diseases, such as depression or neurodegeneration. During the production of beverages and other kinds of food with jujube, such as cake, the peel is usually discarded. Jujube peel extract was found to contain higher amount of phenolic compounds than that of pulp [69]. These findings might imply that jujube peel could serve as an inexpensive source of natural antioxidant to protect neuronal cells against oxidation insult. The seed of jujube is also discarded in either food products or TCM practices. The seed from* Z. jujuba* var.* spinosa*, the sour jujube, possessed hypnotic effect [35, 60], indicating that jujube seed might also be a selection for insomnia as either raw material or source for active ingredient.

The usage of jujube in China is not restricted as a single fruit; it is commonly prescribed in multiherbal decoctions for various purposes. Jujube was commonly employed as the main ingredient within all the herbal formulae written by Zhang Zhongjing (150 BC to 219 AD). According to Zhang's theory of Chinese medicine, jujube was included in herbal decoction as to adjust the taste of other herbs, for example, to harmonize the spleen and stomach, to harmonize the nutrient and defense, and to calm the mind. Having jujube as the active ingredient, the major function of jujube-containing decoctions for the treatment of brain related diseases should be expected.

## Figures and Tables

**Table 1:** Neuronal beneficial properties of jujube.

Findings	Model	Treatment	Reference
Jujube induced neurite outgrowth and neurofilament expression via cAMP-PKA-CREB signaling	Cultured PC12 cells; neuronal differentiation	Jujube extract compared with forskolin and control	[13]
The mature jujube possessed better effect in inducing neurofilament expression than that of the immature one	Cultured PC12 cells; neurofilament expression	Mature jujube extract compared with immature jujube extract	[19]
Jujube stimulated the expressions of neurotrophic factors and antioxidant enzymes	Cultured astrocytes; mRNA expression	Jujube at various concentrations (0–3 mg/ml)	[16]
Jujube protected neuronal cells against oxidation injury via activation of transcriptional activity of ARE	Cultured PC12 cells; tBHP induced oxidative stress	Jujube extract compared with Vit.C, tBHQ^b^, and negative control	[20]
Jujube protected neurons from ischemic damage	Ischemic damage in gerbil hippocampus	Oral administration of jujube extract for 10 days	[21]
Jujube increased pentobarbital-induced sleep time and reduce free movement on mice	Kunming mice, behavior and sleeping studies	Jujube at 8 g/kg was administered orally	[22]
Jujube promoted learning and memory in ovariectomized rat model	SD rats, Morris water maze test	Ovariectomized rats in 6 groups: jujube groups, positive, model, and sham surgery groups	[23]
Hydroalcoholic extract of jujube ameliorates seizures, oxidative stress, and cognitive impairment in epilepsy rat model	Rat, experimental seizure models	Hydroalcoholic extract of jujube (100, 250, 500, and 1000 mg/kg) was administered orally	[24]
Jujube had repairing effects on memory and behavioral disorders produced by NBM lesion in rats	Wistar male rats; Morris water maze test	Rats in 7 groups: normal, AD, AD/normal + jujubes at two doses, and sham	[25]
Methanolic jujube extract activated ChAT. Oleamide from jujube reversed the memory and/or cognitive impairment in mice model	Cultured MC-IXC cells, mice	Oleamide at 0.4–2.4 mM on ChAT^a^ activity; mice were treated with oleamide for 4 weeks	[26]
Jujube increased the cAMP content in plasma and hippocampus of animals	ICR male mice, cAMP level in hippocampus and serum	i.g. administration of jujube at 0.35 g/kg	[27]
A polypeptide Snakin-Z from jujube possessed cholinesterase inhibitory activity	Cholinesterase inhibitory activity	Snakin-Z at 1.5 mg/mL has 80% inhibitory activity	[28]

^a^ChAT, choline acetyltransferase. ^b^tBHQ, *tert*-butyl hydroquinone.
